# Model of Transcriptional Activation by MarA in *Escherichia coli*

**DOI:** 10.1371/journal.pcbi.1000614

**Published:** 2009-12-18

**Authors:** Michael E. Wall, David A. Markowitz, Judah L. Rosner, Robert G. Martin

**Affiliations:** 1 Computer, Computational, and Statistical Sciences Division, Los Alamos National Laboratory, Los Alamos, New Mexico, United States of America; 2 Bioscience Division, Los Alamos National Laboratory, Los Alamos, New Mexico, United States of America; 3 Center for Nonlinear Studies, Los Alamos National Laboratory, Los Alamos, New Mexico, United States of America; 4 Laboratory of Molecular Biology, National Institute of Diabetes, Digestive and Kidney Diseases, National Institutes of Health, Bethesda, Maryland, United States of America; Washington University School of Medicine, United States of America

## Abstract

The AraC family transcription factor MarA activates ∼40 genes (the *marA/soxS/rob* regulon) of the *Escherichia coli* chromosome resulting in different levels of resistance to a wide array of antibiotics and to superoxides. Activation of *marA/soxS/rob* regulon promoters occurs in a well-defined order with respect to the level of MarA; however, the order of activation does not parallel the strength of MarA binding to promoter sequences. To understand this lack of correspondence, we developed a computational model of transcriptional activation in which a transcription factor either increases or decreases RNA polymerase binding, and either accelerates or retards post-binding events associated with transcription initiation. We used the model to analyze data characterizing MarA regulation of promoter activity. The model clearly explains the lack of correspondence between the order of activation and the MarA-DNA affinity and indicates that the order of activation can only be predicted using information about the strength of the full MarA-polymerase-DNA interaction. The analysis further suggests that MarA can activate without increasing polymerase binding and that activation can even involve a *decrease* in polymerase binding, which is opposite to the textbook model of activation by recruitment. These findings are consistent with published chromatin immunoprecipitation assays of interactions between polymerase and the *E. coli* chromosome. We find that activation involving decreased polymerase binding yields lower latency in gene regulation and therefore might confer a competitive advantage to cells. Our model yields insights into requirements for predicting the order of activation of a regulon and enables us to suggest that activation might involve a decrease in polymerase binding which we expect to be an important theme of gene regulation in *E. coli* and beyond.

## Author Summary

When environmental conditions change, cell survival can depend on sudden production of proteins that are normally in low demand. Protein production is controlled by transcription factors which bind to DNA near genes and either increase or decrease RNA production. Many puzzles remain concerning the ways transcription factors do this. Recently we collected data relating the intracellular level of a single transcription factor, MarA, to the increase in expression of several genes related to antibiotic and superoxide resistance in *Escherichia coli*. These data indicated that target genes are turned on in a well-defined order with respect to the level of MarA, enabling cells to mount a response that is commensurate to the level of threat detected in the environment. Here we develop a computational model to yield insight into how MarA turns on its target genes. The modeling suggests that MarA can increase the frequency with which a transcript is made while decreasing the overall presence of the transcription machinery at the start of a gene. This mechanism is opposite to the textbook model of transcriptional activation; nevertheless it enables cells to respond quickly to environmental challenges and is likely of general importance for gene regulation in *E. coli* and beyond.

## Introduction

Transcription factors control cellular protein production by binding to DNA and changing the frequency with which mRNA transcripts are produced. There are hundreds of transcription factors in *Escherichia coli* and while most of these target only a small number of genes, there are several that regulate expression of ten or more genes. Taken together, such global transcription factors directly regulate more-than half of the ∼4,300 genes in *E. coli* and their regulatory interactions yield important insights into the organization of the genetic regulatory network [1],[2],[3]. Because they regulate so many genes, global transcription factors also play a large role in controlling cellular behavior; however, insights into behavior are currently limited by a lack of quantitative information about how transcription factors differentially regulate target genes.

One important global transcription factor is MarA, an AraC family protein that activates ∼40 genes (the *marA/soxS/rob* regulon) of the *Escherichia coli* chromosome resulting in different levels of resistance to a wide array of antibiotics and superoxides (see [4] for references). The effect of MarA at different promoters can vary due to changes in the detailed sequence of the DNA-binding site and its distance from and orientation with respect to the promoter [5],[6]. These variations can influence the order in which the promoters respond to increasing concentrations of MarA and presumably have important functional consequences for *E. coli*.

To characterize quantitative variations in MarA regulation at different promoters, we recently placed the expression of MarA under the control of the LacI repressor, determined the relationship between isopropyl β-D-1-thiogalactopyranoside (IPTG) concentration and the intracellular concentration of MarA, and examined the expression of 10 promoters of the regulon as a function of activator concentration [7]. We found that activation of *marA/soxS/rob* regulon promoters occurs in a well-defined order with respect to the level of MarA, enabling cells to mount a response that is commensurate to the level of threat detected in the environment. We also found that only the *marRAB*, *sodA*, and *micF* promoters were saturated at the highest level of MarA. In contrast with a commonly held assumption, we found that the order of activation does not parallel the strength of MarA binding to promoter sequences. This finding suggested that interactions between MarA and the RNA polymerase transcriptional machinery play an important role in determining the order of activation, but the data did not immediately reveal what the nature of these interactions might be at the various promoters.

Here, we have developed a computational model of promoter activity to understand how interactions between MarA and polymerase activate transcription at the *marRAB*, *sodA*, and *micF* promoters – of the 10 we examined previously, these three promoters are the only ones that exhibited saturation, which provides an important constraint for the modeling. The model was specifically designed to compare a strict recruitment model in which MarA increases polymerase binding but does not increase the rate of post-binding events [8],[9], with a more general model in which activator can either increase or decrease polymerase binding, and can either increase or decrease the rate of post-binding events. For each promoter, we evaluated the agreement of both the strict recruitment model and the general model with the data at many points within a physically reasonable region of parameter space. The model successfully explains why the order of promoter activation does not parallel the strength of MarA-DNA binding. For all promoters, the best fit of the general model was better than that of the strict recruitment model. Comparison to the strict recruitment model and full analysis of the goodness-of-fit landscape suggest that MarA does not increase polymerase binding but does increase the rate of post-binding events at these promoters. Moreover, the analysis for the *micF* promoter suggests that MarA activation can involve a *decrease* in polymerase binding that is associated with low latency in gene regulation. We discuss the broader significance of these findings.

## Results

### Model

Our model choice was tailored to the *in vivo* activity data for the *marRAB*, *sodA*, and *micF* promoters; these data were obtained from batch cultures that were periodically diluted to maintain logarithmic growth [7]. The activity assays were performed after many generations and represent quasi-steady-state levels that are well-matched to a steady-state model of promoter activity. We therefore based our model on a statistical-thermodynamic model that was originally developed to study steady-state transcriptional repression by λ phage repressor [10]. In our model, the promoter exists in a number of distinct states, each of which has a corresponding free energy and activity. The statistical weight of each state in a batch culture ensemble of promoters is given by Boltzmann factors that correspond to thermal equilibrium, and the total promoter activity is calculated as the weighted sum of the individual promoter state activities.

**Figure 1:**
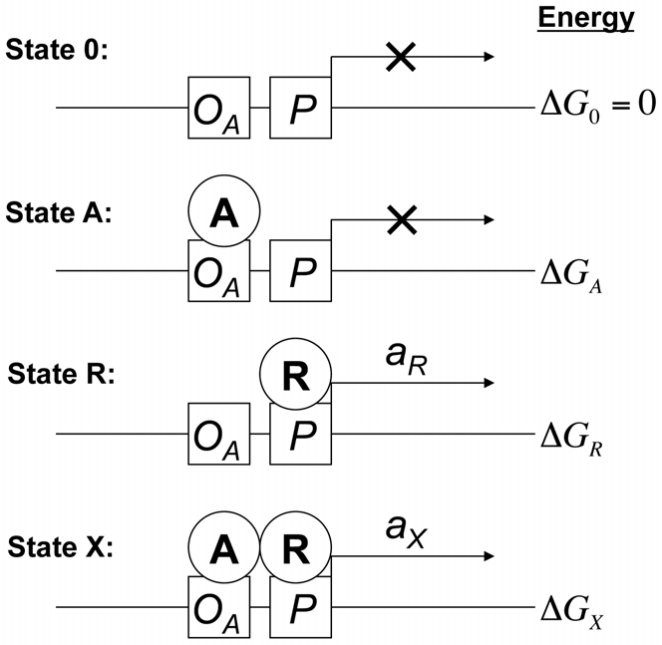
Illustration of promoter states in the model, with corresponding activities and standard free energies.

Our model considers four promoter states enumerated as follows (Fig. 1). In State 0, the promoter is free. This is the reference state with energy 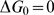 and no activity. In State A, MarA is bound at the operator sequence *O_A_*, yielding free energy 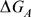 and no activity; in State R, polymerase is bound at the promoter *P*, yielding free energy 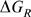 and activity *a_R_*; and in State X, both MarA and polymerase are bound, yielding free energy 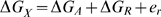, and activity *a_X_*. The term *e_r_* is a recruitment energy that captures the interaction between MarA and polymerase on the DNA: a value *e_r_* = 0 indicates no influence of MarA on the affinity of polymerase, a value *e_r_*<0 indicates that MarA increases the affinity of polymerase, and a value *e_r_*>0 indicates that MarA decreases the affinity of polymerase for the promoter. Unlike a strict recruitment model [11], to enable us to evaluate the likelihood of alternative mechanisms, our model allows for different activities in the presence or absence of MarA, and even allows for the possibility that the promoter activity might be smaller in the presence of MarA.

The free energies of the states with either MarA (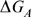) or polymerase (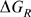) bound are defined for 1 M concentrations of free MarA and polymerase, respectively. These free energies are related to corresponding dissociation constants via 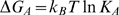 and 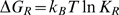 where the dissociation constants *K_A_* and *K_R_* are in molar units. The dissociation constants in turn determine the statistical state weights *p_i_* via the following equations:
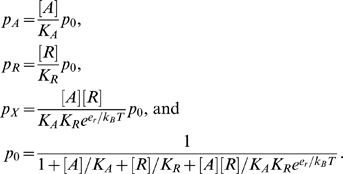
(1)


In Eqs. (1), the first three equations follow from the definition of the dissociation constants and free energies, and the last equation follows from the normalization condition 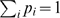.

A novel feature of our model is that it considers the interaction between free MarA and polymerase away from the promoter. This interaction is known to be significant from *in vitro* experimental binding studies [12],[13]; Heyduk et al. [14] found a similar interaction between CRP and polymerase. The equilibrium between free MarA (*A*) and polymerase (*R*) and the MarA-polymerase complex (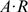) is modeled assuming steady-state equilibration characterized by dissociation constant *K_AR_*:
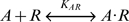
(2)


To account for other interactions such as nonspecific binding of polymerase to DNA, we also let polymerase be sequestered by a background pool of nonspecific binding partners (*B*) with dissociation constant *K_BR_*:
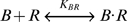
(3)


We assume that interactions with the promoter do not significantly influence the equilibrium. This is a reasonable assumption given that the chromosomal *lacZ* reporter fusions used in Martin et al [7] have a copy number of at most 5 per cell. The model leads to the following equations
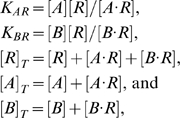
(4)where 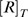, 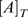, and 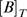 are the total levels of polymerase, MarA, and the background pool in the cell, respectively. Eqs. (4) yield a cubic equation for 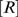 with a positive real root (Text S1). The equation 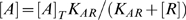 then follows from the first and fourth equations in Eqs. (4). Finally, the expressions for [*R*] and [*A*] may be used to calculate the state weights in Eqs. (1) given values of 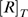, 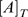, and 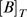.

The total promoter activity is a weighted sum of the activities in each state. No transcription occurs in states 0 or A, in which polymerase is absent from the promoter. Transcription occurs in state R with activity *a_R_*, and in state X with activity *γa_R_*; polymerase is present at the promoter in both of these states. The equation for the total activity *a* is therefore
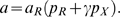
(5)


Eq. (5) represents the general promoter activity model; in the strict recruitment limit, the value of *γ* is equal to 1 indicating that polymerase activity is the same in the presence vs. the absence of MarA. We assume that the total promoter activity *a* in Eq. (5) is proportional to the measured β-galactosidase activity resulting from *in vivo lacZ* reporter expression.

### Calibration of IPTG against MarA

We calibrated IPTG levels against MarA levels using analyses of Western blots in multiple lanes from a single gel [7]. Such calibration is rarely performed even in highly quantitative studies of gene regulation; however, here the calibration is the key to enabling the mechanistic insights that we sought in the modeling. The MarA vs. IPTG data are well-described using the equation
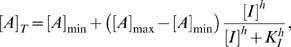
(6)where [*I*] is the extracellular IPTG concentration, [*A*]*_T_* is the total cellular MarA concentration that appears in Eqs. (4), 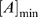 = 20 molecules cell^−1^, 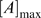 = 20,486 molecules cell^−1^, *K_I_* = 18.98 µM, and *h* = 2.46 (Figure S1). Due to errors in quantifying small MarA levels, we were unable to obtain a good estimate of 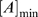 from the data; however, we believe that there is some expression of MarA from the plasmid in the absence of IPTG because the basal activity of the *lacZ* fusions is slightly higher in cells carrying the MarA plasmid than in cells carrying a control plasmid. The value 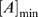 = 20 molecules cell^−1^ is consistent with the 1,000-fold induction of the wild-type *lac* system and yields reasonable fits to the data. To account for differences between the plasmid expression system and the wild-type system, we tried values as high as 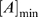 = 200 molecules cell^−1^; however, such models agreed poorly with the promoter activity data. We therefore used 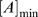 = 20 for the modeling studies described below.

### Simulation of promoter activity profiles

The experimental data consist of measurements of β-galactosidase activity coupled with standard errors at defined concentrations of external IPTG (Table S1). To model the data for a given promoter, simulated activity profiles were obtained by calculating the activity at each IPTG concentration using Eqs. (1), (4), and (5). Values of *K_AR_*, *K_BR_*, *K_A_*, *K_R_*, *e_r_*, [*B*]*_T_* and [*R*]*_T_* were sampled from allowed ranges defined with guidance both from the literature and by our measurements (Methods), and values of [*A*]*_T_* for each IPTG level were obtained using Eq. (6) which was constrained by the calibration. Values of *p_R_* and *p_X_* were then calculated using Eqs. (1) and (4).

The weights *p_R_* and *p_X_* determine the activity values through Eq. (5), which includes additional parameters *a_R_* and *γ*. The values of these parameters may vary among promoters. To simulate promoter activity for a strict recruitment model, the value of *γ* was set to 1, and linear regression was used to find the value of *a_R_* in Eq. (5) that minimized a standard χ^2^ goodness-of-fit statistic calculated between the simulated and measured activity values. To simulate promoter activity for the more general model of activation, we performed a linear regression to simultaneously find the best-fit values of *a_R_* and *γ*. To further sample the fitting landscape, we then randomly sampled five values of *γ* that differed from the optimum by up to a factor of 100, finding the best-fit value of *a_R_* in each case.

### Modeling approach

At this point it would be typical to seek the combination of parameter values that minimize the value of χ^2^ and draw some conclusions based on the resulting best-fit model. However, we were concerned about the possibility that many combinations of parameter values might yield reasonable (if not optimal) fits to the data and therefore adopted a more rigorous modeling approach. We note that this concern did not come from comparing the number of data points to the number of parameters: the model has 9 parameters, whereas we made multiple measurements of each promoter's activity at 10 or more different IPTG levels (Table S1). This is adequate to constrain a fit. Rather, our concern was that all of the measured activation profiles have a similar S shape that might be described using ∼4 parameters (minimal activity; maximal activity; IPTG level at the midpoint; and slope in the regulatable region), suggesting that our 9-parameter model might reasonably fit the data for a wide range of parameter values.

Instead of drawing conclusions based on the properties of a single best-fit model, we therefore sought more robust results by adopting a Bayesian approach (Methods). In our approach, we began by defining a range of physically reasonable parameter values for *K_AR_*, *K_BR_*, *K_A_*, *K_R_*, *K_X_*, [*B*]*_T_*, and [*R*]*_T_*, and randomly sampled a large number (10,000 or more) of combinations of parameter values from within the allowed range (Methods; Table 1). For each such combination, as described above, we explored values of *γ* and *a_R_* using either a strict recruitment model or a more general promoter activity model. (In practice, we found that a certain fraction of the parameter value combinations samples yielded unphysical models in which activation required a negative value of *γ*; these samples were removed in the analysis.) We treated the resulting χ^2^ as an indicator for the quality of a model and used it to define a goodness-of-fit landscape in parameter space. Sampling the landscape in this way permitted us to identify entire regions of parameter space that correspond to reasonable models, and to further determine whether models within the identified region share common mechanisms of activation. This approach therefore enables a much more robust suggestion of activation mechanisms than would conclusions drawn by examining the properties of a single best-fit model.

**Table 1:** Parameter values used to model activation of *marRAB*, *sodA*, and *micF* promoters by MarA.

Parameter	*marRAB*	*sodA*	*micF*
*K_AR_* [µM]	0.3, 1.0, 10, 21^a^, 100	0.3, 1.0, 10, 21^a^, 100	0.3, 1.0, 10, 21^a^, 100
*K_A_* [nM]	75^a^, (0.25–2,500)	2,000^a^, (0.25–2,500)	50^a^, (0.25–2,500)
*K_R_* [nM]	(1–10,000)^a^; (100–10^6^)	(1–10,000)^a^; (100–10^6^)	(1–10,000)^a^; (100–10^6^)
*e_r_/k_B_T*	(−4.6–+4.6)^a^	(−4.6–+4.6)^a^	(−4.6–+4.6)^a^
[*R*]*_T_* [Molec cell^−1^]	1,000; 3,000^a^	1,000; 3,000^a^	1,000; 3,000^a^

a Nominal parameter values used to create Figs. 3–5.

### The general activation model yields better fits than the strict recruitment model

**Figure 2:**
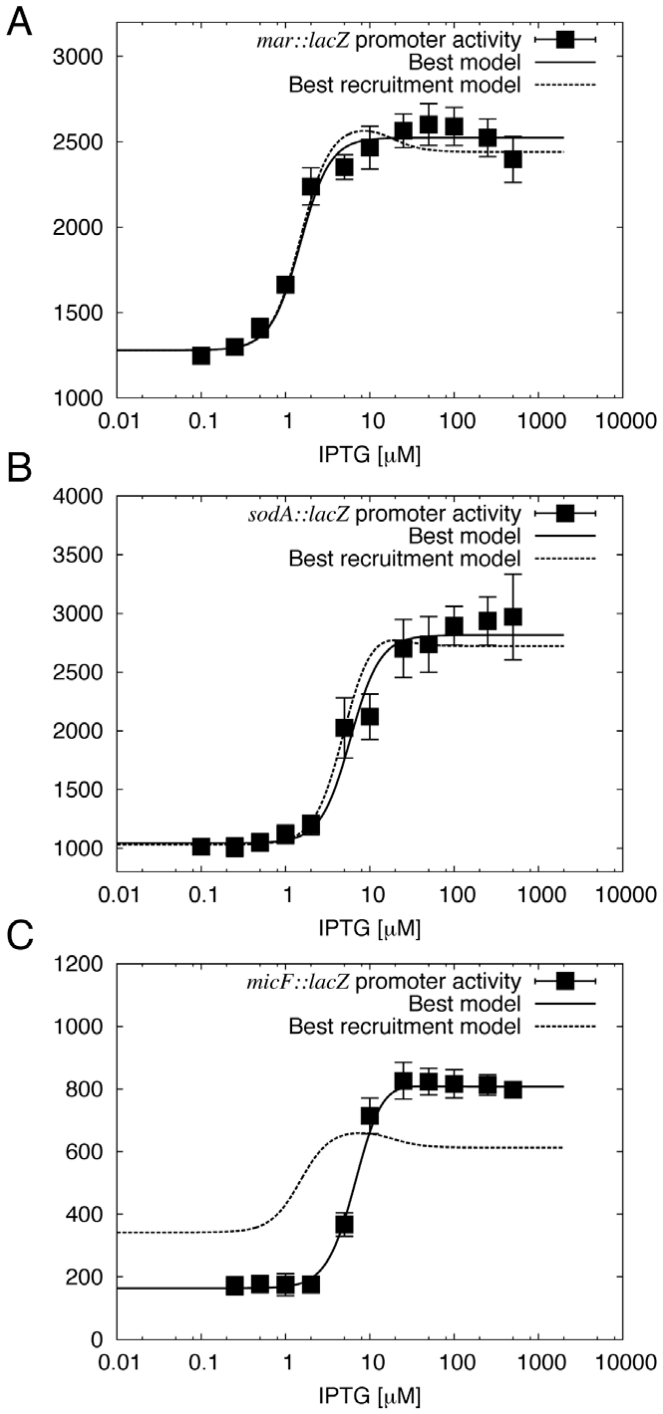
Fits of the best models of promoter activity. A) *marRAB*; B) *sodA*; C) *micF*. Error bars for the data correspond to the standard error of the mean calculated from multiple trials (Table S1). Corresponding χ^2^ and parameter values are given in Table 2.

The best-fit activity profiles for the models of each promoter are illustrated in Fig. 2; the parameters of these models are listed in Table 2. The quality of the fits indicates that the general activation model is entirely consistent with the observed IPTG-dependent activity of the *marRAB*, *sodA* and *micF* promoters: the χ^2^ values of these fits are 9.15, 6.72, and 2.49, respectively. The strict recruitment model yielded larger χ^2^ values of 14.43, 11.33, and 622.3, respectively. Overall, the general activation model was more consistent with the promoter activity data; in particular, the strict recruitment model was inconsistent with the *micF* data whereas the general model was consistent with these data. Table 2 also includes asymmetric errors (Methods) that indicate the degree to which parameter values are constrained by the data. These errors indicate that parameter values of the *micF* model are well-constrained compared to parameter values for the *marRAB* and *sodA* models. The magnitude of these errors suggests that analysis of just the best-fit model would not yield robust conclusions concerning mechanisms of activation: for example, the best-fit value of *e_r_* for the *marRAB* model is −0.44 *k_B_T*, but the span of the error includes positive values of *e_r_*. In the following sections, rather than relying on analysis of the best-fit model, we use analysis of the full fitting landscape to suggest mechanisms of activation of these promoters.

**Table 2:** Properties of models with the lowest value of χ^2^.

	*marRAB*	*sodA*	*micF*
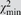	9.15	6.72	2.49
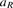	5.79(+1084)(−4.43)	1017(+6357)(−30)	157(+8)(−9)
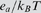	−6.08(+2.79)(−2.33)	−1.06(+0.50)(−4.00)	−6.21(+2.57)(−0.29)
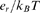	−0.44(+0.70)(−0.59)	+0.37(+4.38)(−0.81)	+8.94(+0.12)(−2.67)
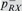	0.995(+0.001)(−0.334)	0.996(+0.003)(−0.493)	0.979(+0.007)(−0.005)
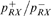	1.00(+1.87)(−0.17)	0.998(+1.049)(−0.681)	0.089(+0.264)(−0.014)

Parameter values were sampled using nominal values and ranges in Table 1. Values of *x*_min_ are listed with asymmetric errors σ*_+x_* and σ*_−x_* as *x*_min_(*+σ_+x_*)(−σ*_−x_*) (errors are defined in Eq. (11)).

### MarA accelerates polymerase kinetics

To determine whether polymerase activity increases or decreases when MarA is bound to the promoter, we analyzed the parameter *γ*, which is equal to the ratio of polymerase activity in the presence vs. the absence of activator (Eq. (5)). It is convenient to perform the analysis using the acceleration energy, *e_a_*, defined as
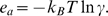
(7)


The acceleration energy defined in Eq. (7) is equivalent to the activator-induced change in the activation energy of a lumped transcription initiation process, under the assumption that initiation follows an Arrhenius law with the same attack frequency in the presence or absence of activator. A value *e_a_* = 0 corresponds to an unchanged polymerase activity; this condition is consistent with a strict recruitment model of transcriptional activation, in which activator increases the occupancy of polymerase at the promoter but does not alter polymerase activity [8],[9]. Models with *e_a_*<0 exhibit acceleration and models with *e_a_*>0 exhibit retardation of polymerase activity in the presence of activator.

**Figure 3:**
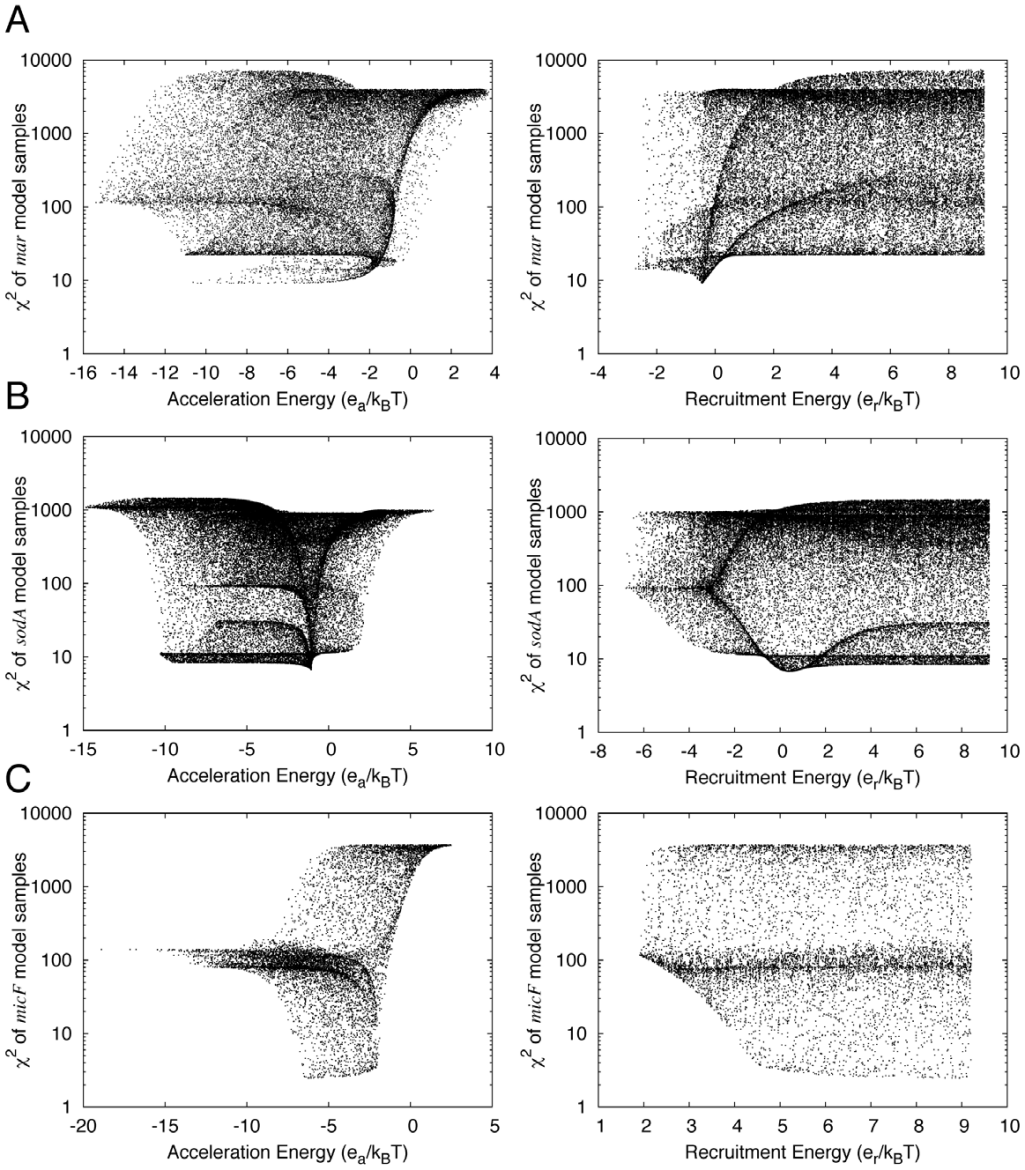
Dependence of χ^2^ of promoter activity models on the acceleration energy and recruitment energy. A) *marRAB*; B) *sodA*; C) *micF*. The value of χ^2^ is plotted on the *y*-axis in all panels. The left panels plot acceleration energy (*e_a_*) and right panels plot recruitment energy (*e_r_*) on the *x*-axis. Points correspond to 10,000 different sets of parameter values, sampled using the nominal values in Table 1. Points with the lowest χ^2^ value correspond to the systems in Table 2.

**Figure 4 pcbi-1000614-g004:**
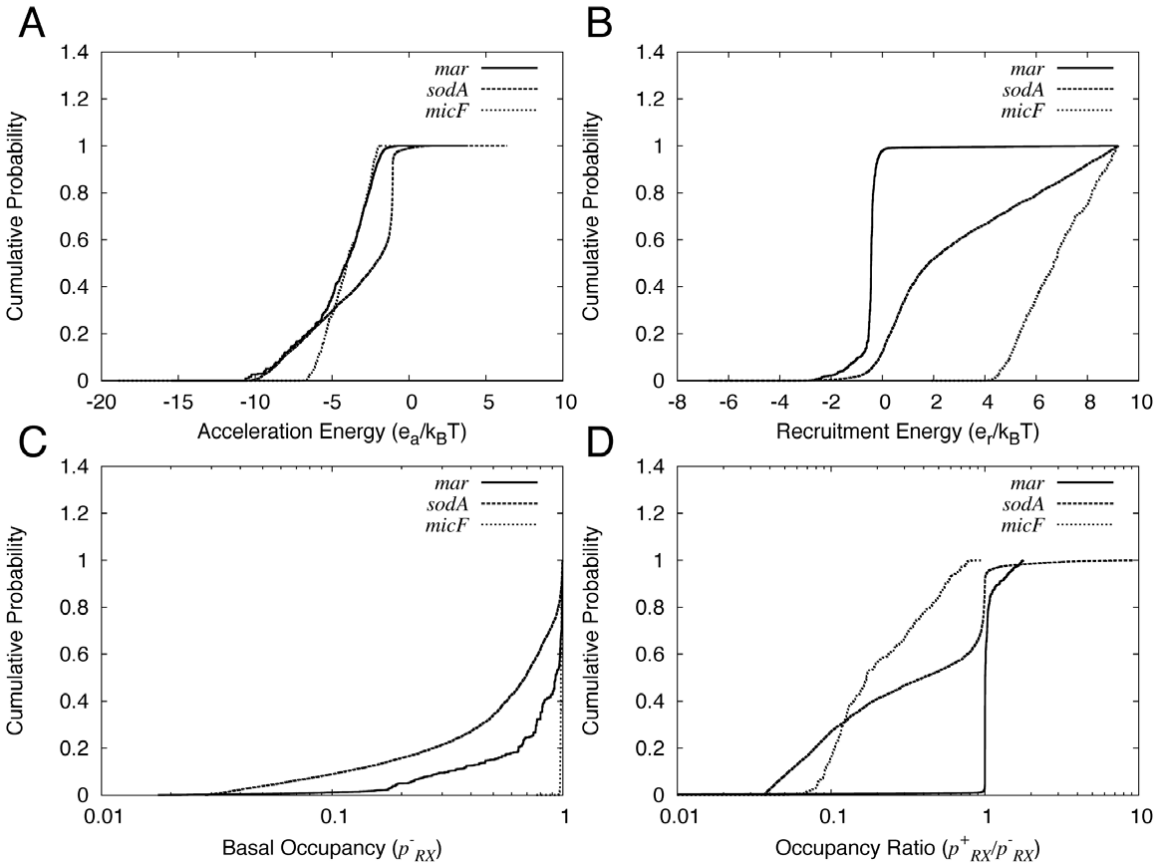
Cumulative probability distribution functions used to interpret the modeling results. The panels show plots of *C*(*x*), where the *x*-axis corresponds to A) acceleration energy; B) recruitment energy; C) basal occupancy of polymerase at the promoter; and D) polymerase occupancy ratio in the presence vs. absence of MarA. Results for *marRAB* (solid line), *sodA* (dashed line), and *micF* (dotted line) are plotted together in each panel.

For each promoter, the model with the lowest χ^2^ value has a negative acceleration energy (Table 2). Scatter plots of χ^2^ vs. *e_a_* for parameter samples indicate that other models with low χ^2^ values also tend to have negative acceleration energies (Fig. 3, left panels). To quantify this trend, we used Bayesian methods to estimate cumulative distribution functions *C*(*e_a_*) for the posterior probability of *e_a_* values (Methods). (It is important to keep in mind that these distributions do not indicate absolute probabilities as their calculation entails certain assumptions about the likelihood function and the prior distribution of parameter values (Methods); nevertheless, given these assumptions, the distributions provide a valuable means of interpreting the modeling results.) The distributions indicate that nearly all of the density lies within the region *e_a_*<1 (Fig. 4A): the value of the distribution function at *e_a_* = 0 is essentially 1 for the *marRAB* and *micF* models, and is 0.99 for the *sodA* model. The modeling therefore suggests that activator increases polymerase activity at the *marRAB*, *sodA*, and *micF* promoters.

### MarA increases polymerase affinity but not occupancy at the *marRAB* promoter

To determine whether the affinity of polymerase for the promoter changes in the presence vs. the absence of MarA, we analyzed the recruitment energy *e_r_*. As mentioned above, a value *e_r_* = 0 indicates no influence of MarA on the affinity of polymerase, a value *e_r_*<0 indicates that MarA increases the affinity of polymerase, and a value *e_r_*>0 indicates that MarA decreases the affinity of polymerase for the promoter.

For *marRAB*, the model with the lowest χ^2^ has *e_r_* = −0.44 *k_B_T* (Table 2). A scatter plot indicates that other models with low χ^2^ tend to have negative values of *e_r_* (Fig. 3A, right panel). The cumulative distribution function *C*(*e_r_*) also shows that most of the probability density corresponds to negative values of *e_r_* (Fig. 4B): the value is 0.978 at *e_r_* = 0. The modeling therefore suggests that MarA activation of *marRAB* involves an increase in the affinity of polymerase at the promoter.

**Figure 5:**
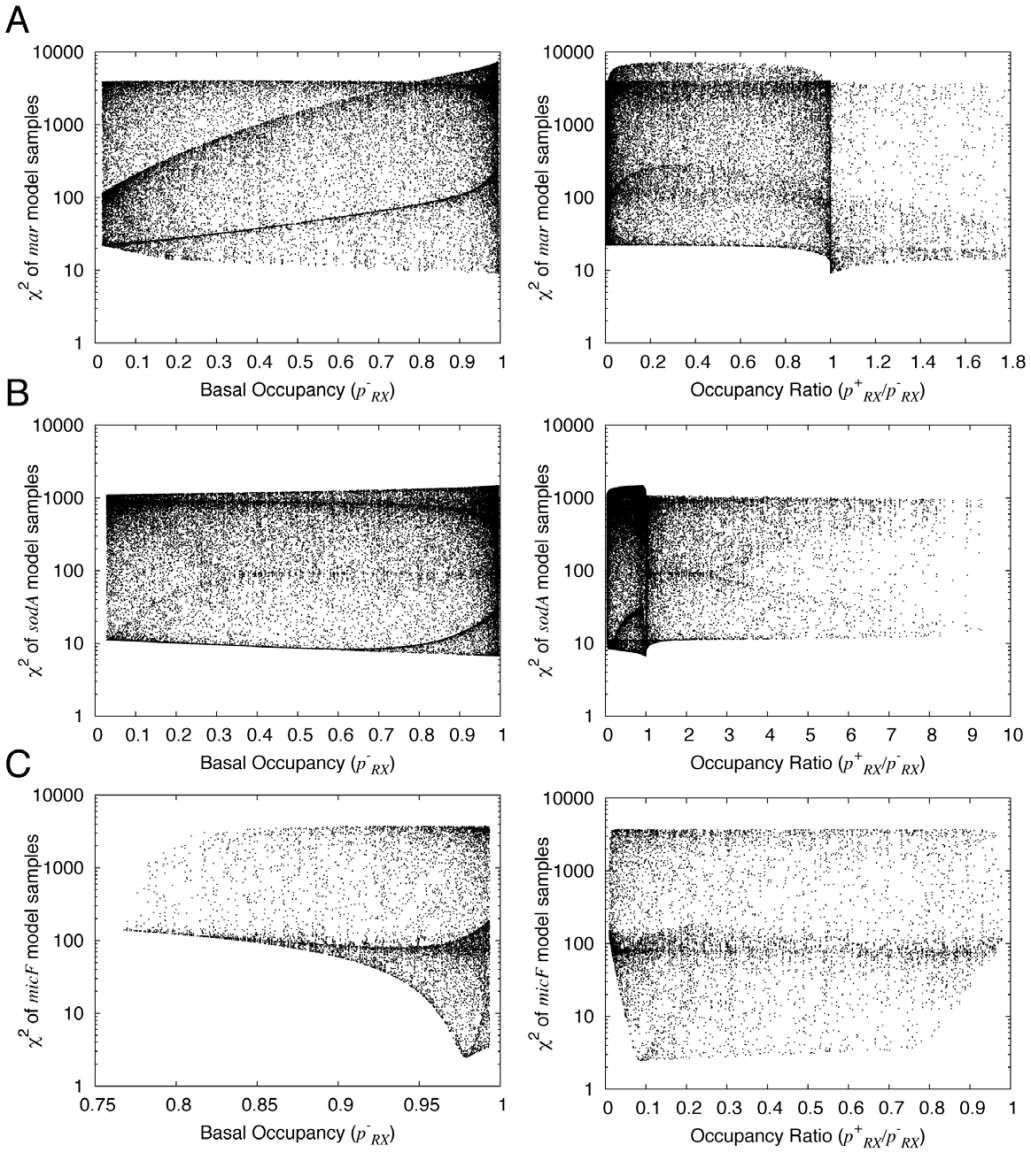
Dependence of χ^2^ of promoter activity models on the basal occupancy of polymerase at the promoter, and the occupancy ratio in the presence vs. the absence of MarA. A) *marRAB*; B) *sodA*; and C) *micF*. The value of χ^2^ is plotted on the *y*-axis in all panels. The left panels plot the basal occupancy (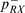) and right panels plot the occupancy ratio (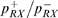) on the *x*-axis. Parameter values were sampled as for Fig. 3, using the nominal values in Table 1.

It is important to note that an increase in affinity of polymerase for the promoter does not always translate into a significant increase in occupancy. For example, if polymerase is already bound with essentially unit occupancy in the absence of activator, even a large increase in affinity will result in an insignificant increase in occupancy. We therefore analyzed and compared the total occupancy of polymerase at the promoter,
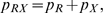
(8)at low (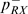) and high (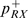) levels of MarA. For *marRAB*, the basal occupancy 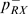 for the best-fit model is 0.995, and the occupancy ratio 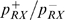 is 1.00. Scatter plots indicate that the fits are relatively insensitive to the precise value of 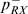 (Fig. 5A, left panel), but that the low-χ^2^ values of 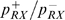 are more sharply centered on 1.00 (Fig. 5A, right panel). The cumulative distributions quantify these trends: in 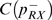, the cumulative probability increases slowly and steadily from about 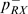 = 0.1 all the way to 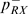 = 1.0, and half of the probability density lies below 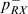 = 0.93 (Fig. 4C). In 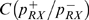, there is little density below 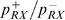 = 1.0, the distribution increases sharply in the neighborhood of 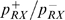 = 1.0, and 79% of the density lies below 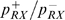 = 1.05 (Fig. 4D). Overall, the model does not strongly indicate whether polymerase is bound or unbound at the promoter in the absence of MarA but it does weakly suggest that MarA does not increase the occupancy of polymerase at the promoter.

### MarA decreases both polymerase affinity and occupancy at the *sodA* and *micF* promoters

For both *sodA* and *micF*, the models with the lowest χ^2^ have *e_r_*>0 (Table 2). The scatter plot for *sodA* indicates that other low χ^2^ models also tend to have positive values of *e_r_* (Fig. 3B, right panel). In the case of *micF*, the scatter plot indicates that *all* models have positive *e_r_*. This requires some explanation, as our sampling did produce a roughly equal number of models with positive and negative *e_r_*. As mentioned above, some of the parameter value combinations were eliminated because they yielded unphysical models with a negative optimal value of *γ*. This is the reason for the different number of points that is apparent for different promoters in Figs. 3 and 5. In the case of *micF*, the number of unphysical samples was especially high, and included all those with negative *e_r_*.

The cumulative distributions *C*(*e_r_*) for these promoters support the trends seen in the scatter plots (Fig. 4B). In the case of *sodA*, 88% of the density lies within *e_r_*>0. In the case of *micF*, all of the density lies within *e_r_*>0. Activation in this model therefore involves a decrease in the affinity of polymerase for both the *sodA* and *micF* promoters.

Analysis of 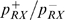 and 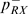 suggest that binding of MarA decreases the occupancy of polymerase at both *sodA* and *micF*. In the case of *sodA*, the best-fit model is misleading, as 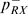 = 0.996 and 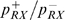 = 0.998 for this model (Table 2), suggesting no influence of activator on polymerase occupancy. However, scatter plots show that the value of 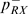 is poorly constrained (Fig. 5B, left panel), and that there are many low-χ^2^ models with 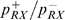
<1 (Fig. 5B, right panel). These observations are supported by the cumulative distributions 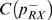 and 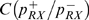: there is a relatively steady increase in 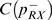 between 0.03 and 1 (Fig. 4C), and 89% of the 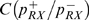 distribution lies within 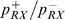
<1, with 70% below 0.95 (Fig. 4D). In the case of *micF* the results are more clear-cut: all physically reasonable models have 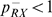 and 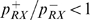 (Figs. 4C, 4D, and 5C).

### Results are robust to parameter variation

In addition to the nominal parameter variations in Table 1, we examined the sensitivity of the results to wider parameter variation (Methods). The variations explored were: changing the value of [*R*]*_T_* to 1,000 copies per cell; changing the value of *K_AR_* to 0.3, 1, 10, and 100 µM; and, instead of fixing the value of *K_A_* for each promoter, sampling this parameter randomly between 0.25–2,000 nM. We also repeated the entire analysis, including these variations, using [*B*]*_T_* = 0 which eliminates the sequestering of free polymerase by other interaction partners. Thus, the above sampling and analysis approach was applied 13 additional times for each of the promoters. For the general model, all of these variations still yielded at least some promoter activation curves with reasonable values of χ^2^. With the exception of one variation, the general model yielded significantly better fits than the strict recruitment model for all promoters. The only exception was *K_AR_* = 100 µM, which yielded best-fit strict recruitment models of *marRAB* (χ^2^ = 9.67) and *sodA* (χ^2^ = 8.02) that were similar in quality to the general model; however, this was not so for *micF* (χ^2^ = 536), and this value of *K_AR_* is at least a factor of five higher than the values measured *in vitro* [12],[13]. Using [*B*]*_T_* = 0 yielded only poor fits in the strict recruitment limit (e.g., χ^2^ values of 542, 63.8, and 1071 for the best-fit models of *marRAB*, *sodA*, and *micF* using otherwise nominal parameter values from Table 1), and favored models of *sodA* in which MarA does not change polymerase occupancy.

### Further validation of the model using CRP activation data

Given the results obtained for the MarA activation data, we wondered whether our model would yield expected results when applied to a transcription factor that is known to increase the occupancy of polymerase at target promoters. We therefore further validated the model by analyzing published data on transcriptional activation of the *lac* operon by cAMP-CRP [15]. The cAMP-CRP-dependent relative promoter activity was represented using the equation 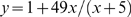, where *x* is the concentration of the active CRP dimer in nM; this expression is consistent with the data published in Bintu *et al.* [15]. We used a strict recruitment model with *γ* = 1, *K_A_* = 5 nM, *K_AR_* = 0.3 µM [14], and parameters otherwise the same as the nominal values in Table 1. Consistent with expectations [9], we found that the recruitment model was entirely consistent with the CRP-dependent *lac* promoter activity (Figure S2). Thus, our modeling method is able to distinguish situations where recruitment applies (e.g., *lac*) from those described here where it does not apply.

## Discussion

The major conclusion of our study is that transcriptional activation can involve a decrease in polymerase binding at the promoter. Our model specifically predicts this is the case for MarA activation of *sodA* and *micF*. The model also predicts that MarA does not increase the occupancy of polymerase at the *marRAB* promoter. For all of these promoters, the model predicts that activation occurs largely through an increase in polymerase activity when MarA is bound.

These predictions are consistent with two genome-wide studies of polymerase interactions with the *E. coli* chromosome [16],[17]. Grainger and coworkers [16] reported detection of polymerase at the *sodA* promoter but not the *marRAB* promoter; that study did not consider interactions at the *micF* promoter which controls expression of an antisense mRNA transcript. In addition, we cross-referenced the oligonucleotide coordinates of Herring *et al.* [17] to transcriptional start sites annotated in the EcoCyc database [18], and found strong-binding 50 bp DNA sequences correctly positioned with respect to *sodA* (sequence beginning at 4,098,720 upstream of 4,098,780 start) and *micF* (sequence beginning at 2,311,050 upstream of 2,311,106 start), but not *marRAB* (only weakly binding sequences near 1,617,117 start). The presence of polymerase at *sodA* and *micF* in uninduced cells strongly suggests that an increase in the affinity of polymerase is not needed to activate these promoters which is consistent with the mechanisms of MarA activation suggested here.

Although the possibility of activation involving decreased polymerase binding might at first seem surprising, a decrease in polymerase binding should really be seen as a natural consequence of accelerated polymerase kinetics. Using a Michaelis-Menten equation to describe transcription initiation with a lumped forward rate *k_f_*, the value of *K_M_* = (*k_off_*+*k_f_*)/*k_on_* increases when *k_f_* increases and the polymerase binding and dissociation rates *k_on_* and *k_off_* remain constant. Thus, the apparent affinity of polymerase for the promoter decreases when the forward rate of the reaction increases. Along these lines, it is also important to note a second potentially counterintuitive possibility: activation might involve retardation of polymerase kinetics when accompanied by a sufficiently large increase in polymerase binding. This is also a natural association because an attraction between activator and polymerase at the promoter has the potential to hinder clearance.

Mechanisms of transcriptional activation have long been an important subject of research and debate. The textbook mechanism for activation of σ^70^ promoters is recruitment [8],[9],[19], in which activator merely increases the binding of RNA polymerase at the promoter [20], and the classic example is activation of the *lac* operon by cAMP-CRP [8]. The simplicity of the recruitment model is appealing; however, it has long been known that transcriptional activation by λ phage repressor can occur through acceleration of post-binding events leading to promoter clearance [21],[22],[23], and that it is even possible for an activator to directly stimulate polymerase transcription without binding to DNA [24]. In addition, σ^54^-polymerase binds at promoters and is activated by enhancers that utilize nucleotides to melt DNA, leading to open complex formation [25]. This use of enhancers in activation of σ^54^-polymerase is reminiscent of activation of stalled polymerase in eukaryotes [26]; the similarity is limited, however, since polymerase stalling in eukaryotes occurs after transcription has already begun [27]. Aspects of the interplay between the energetics of binding and post-binding events and how they govern regulation of transcription have been examined previously [28]. In this regard, the main novel outcome of our work is not the finding that mechanisms other than increasing polymerase binding might be important for transcriptional activation, but rather the suggestion that activation might involve a decrease in polymerase binding.

Another important outcome of our work is a model that explains why the strength of MarA binding to promoters does not parallel the order in which promoters are activated with increasing MarA. A critical feature of our model in this respect is explicit consideration of polymerase interactions with MarA and the promoter. Because of these interactions, the shape of the activation profile is not merely governed by *K_A_*, the MarA-DNA dissociation constant, but is strongly influenced by *e_r_* which characterizes the interaction between DNA and the MarA-polymerase complex (Eqs. (1)). The model therefore quite generally indicates that the strength of activator binding is not expected to parallel the order of activation. This finding not only runs counter to common assumptions in modeling of gene regulation, but also has important implications for prediction of regulon behavior, *i.e.*, one cannot expect to predict the order of regulon activation *in vivo* by measuring the affinity of activator for promoter DNA sequences *in vitro*. By contrast, we expect interactions with polymerase to be less important when a repressor decreases expression by interfering with polymerase binding at the promoter. Such interference corresponds to very large values of *e_r_* in our model which increases the importance of *K_A_* in determining the promoter activity profile (Eqs. (1)). As a consequence, we expect that it might be possible to exploit *in vitro* DNA-binding data to predict the order of *repression* (or derepression) of a regulon.

It is important to note that our model was developed using data from *marRAB*- *rob-* strains [7], in which the repressor MarR is absent. In wild-type *E. coli*, MarR not only blocks polymerase binding but also blocks MarA binding at *marRAB* [29]. We therefore do not expect polymerase to bind at the *marRAB* promoter in the absence of inducers that relieve MarR repression. On the other hand, in wild-type *E. coli*, we do expect polymerase to be bound at the *sodA* and *micF* promoters in the absence of inducers, as supported by the chromatin immunoprecipitation experiments cited above [16],[17]. Rob is also missing in the *marRAB*- *rob-* strains. Rob is constitutively expressed [30],[31] and might recruit polymerase to the *sodA* and *micF* promoters. However, in the absence of inducers, such as dipyridyl, which bind to Rob and stimulate activation of target promoters [32], Rob is mostly sequestered in inclusion bodies [33] and cannot access the DNA [34]. Therefore evidence exists that polymerase binds at the *sodA* and *micF* promoters in wild-type cells without recruitment by Rob or MarA.

Finally we note that activation involving a decrease in polymerase binding decreases latency in both activation and de-activation of gene expression. In the case of activation, as noted in the above argument assuming Michaelis-Menten reaction kinetics, the decrease in polymerase binding is associated with acceleration through an increase in the rate of transcription initiation. In the case of de-activation, a decrease in polymerase binding is associated with acceleration through an increase in the polymerase off rate. Decreases in gene regulation latency can confer a competitive advantage to *E. coli* in an ecological context [35]. We therefore expect activation involving a decrease in polymerase binding to be an important theme of gene regulation in *E. coli* and beyond.

## Methods

### Parameter values

We used a wide range of parameter values to model the MarA-dependent activity of the *marRAB*, *sodA*, and *micF* promoters (Table 1). These values were obtained as follows:

#### *K_AR_*

Using a liquid chromatography assay, Martin *et al.* [13] measured a 0.3 µM dissociation constant for MarA-polymerase complex formation in a crystallization buffer. Dangi *et al.* [12] obtained a value of 21 µM in low-salt conditions using NMR. Because we consider the NMR measurement to be more reliable, we selected a nominal value of 21 µM for *K_AR_*. However, we are uncertain about the correct value to use *in vivo*. To account for uncertainty in *K_AR_*, we explored values of 0.3 µM, 1.0 µM, 10 µM, and 100 µM. We expect the value of *K_AR_* to be promoter-independent, and therefore only compare models across promoters using the same value of *K_AR_*.

#### *K_A_*

The nominal value of 75 nM for the MarA-*mar* promoter dissociation constant was obtained from the gel retardation assay in Martin *et al.* [13]. The nominal value of 2,000 nM for *sodA* was chosen to be consistent with the lack of binding observed in Martin *et al.* [13]. The nominal value of 50 nM for *micF* was chosen from a range of measured values from 8 nM to 80 nM, depending on the preparation (R.G. Martin, unpublished results). To determine whether the qualitative conclusions about activation mechanisms are sensitive to the particular value of *K_A_*, we also analyzed the model using a wide range of values for each promoter, 0.25–2,500 nM.

#### *K_R_*

The value of the dissociation constant for polymerase binding to the promoter is unknown and can vary depending on the promoter. Marr & Roberts [36] measured a dissociation constant of 3 nM for the σ^70^ holoenzyme binding to a 19 bp oligonucleotide containing the TATAAT consensus sequence. For the nominal ranges in Table 1, we analyze models with a range of values from 1 nM (strong binding) to 10,000 nM (weak binding). For the recruitment model, following the expectation that polymerase is bound with less than unit occupancy in the absence of MarA, we considered a higher range of values extending from 100 nM to 10^6^ nM.

#### *e_r_*

The free energy of interaction between MarA and polymerase at the promoter is unknown and can vary depending on the promoter. We found reasonable fits by analyzing models in which *e_r_* varies from −4.6 *k_B_T* to 4.6 *k_B_T*.

#### [*R*]*_T_*

Ishihama [37] and Meuller-Hill [38] estimate the total number of polymerase molecules in the *E. coli* cell at 2,000 and 3,000, respectively. Although *marRAB*, *micF*, and *sodA* are σ^70^ promoters, polymerase is distributed among holoenzymes that contain different σ factors in *E. coli*. We used a nominal value of 3,000 copies per cell, and analyzed the sensitivity of the fits to a smaller value of 1,000 copies per cell.

#### *K_BR_* and [*B*]*_T_*

Similar to Bintu et al. [11], we assume that polymerase binds nonspecifically to DNA with dissociation constant *K_BR_* = 10 µM. However, instead of simply using a value of 5×10^6^ binding sites (the approximate number of base pairs in the *E. coli* chromosome), we allow for diffusion of polymerase on DNA between binding and unbinding events which decreases the effective number of sites. Guided by single-molecule studies of this process for *lac* repressor [39],[40], we assume polymerase diffuses about 50 bp between binding and unbinding, yielding a reduced estimate of [*B*]*_T_* = 10^5^ sites. In our models, these values of *K_BR_* and [*B*]*_T_* cause polymerase levels to be buffered, keeping the level nearly unchanged at about 100 copies per cell when [*R*]*_T_* = 3,000 copies per cell, even as the concentration of MarA increases and polymerase is diverted into the MarA-polymerase complex. We also consider the sensitivity of our results to this effect by eliminating it altogether, setting [*B*]*_T_* = 0.

#### *a_R_* and *γ*

For each sample, we evaluated the model in the strict recruitment limit, *γ* = 1, and found the value of *γ* that minimized the value of χ^2^ in the general model. We then randomly sampled five additional values of *γ* within a factor of 100 of the optimum. The scale factor *a_R_* captures numerous physical effects and was always calculated to minimize the value of χ^2^. Optimal models were found by linear regression using Eq. (5).

For each model of each promoter, we randomly sampled 10,000 sets of parameter values from the nominal ranges in Table 1 and calculated simulated IPTG-dependent activation profiles. The strict recruitment model only used the value *γ* = 1. For the general model, aside from the optimal value of *γ*, we sampled five additional values. Thus, 10,000 sets of parameter values were sampled for each strict recruitment model and 60,000 sets of parameter values were sampled for each general model of promoter activity. Parameter values were sampled in a log uniform manner except for *e_r_*, which was sampled linearly. We explored a wider range of parameter values as described in the manuscript; we used the above sampling scheme for each of these variations. Fits were evaluated using a standard χ^2^ statistic.

### Analysis of fitting landscapes

The values of χ^2^ determined for different parameter value combinations represent samples in a fitting landscape. We used Bayesian methods to analyze the fitting landscape, assuming a likelihood function 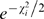 for a sample with parameter combination *i*. In using this likelihood function, we assume that the errors in measurements of mean promoter activity are independent and normally distributed with widths equal to the standard error of the mean (error values in Table S1). The probability *P_i_* of the sample *i* given the data is estimated as
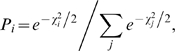
(9)where the index *j* is summed over all samples. The cumulative distribution function *C*(*x*) for a parameter *x* is then given by
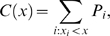
(10)where *x_i_* is the value of parameter *x* in sample *i*, and the sum is restricted to samples *i* where *x_i_*<*x*. *C*(*x*) is interpreted as an estimate of the probability that the parameter has a value less than *x*, given *all* of the assumptions of the modeling, including the sampling scheme.

To quantify the degree of uncertainty in estimated parameter values within the nominal range, we calculated asymmetric errors of parameter values with respect to the optimum (Table 2). The squared errors for parameter *x* were calculated using the equation
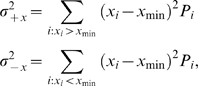
(11)where *x*_min_ is the value of *x* in the sample with the lowest value of 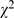.

## Supporting Information

Figure S1 Calibration of IPTG levels against MarA levels. The data (boxes) are well-described by Eq. (6) (line).
(0.02 MB PDF)

Figure S2 Fit of the recruitment model to CRP-dependent activity of the *lac* promoter. The data were generated using a Hill equation based on previously measured promoter activity data from Ref. [15], and the error bars were arbitrarily assigned for the fitting.
(0.02 MB PDF)

Table S1 Promoter activity data.
(0.07 MB PDF)

Text S1 Equation for the free polymerase concentration obtained by solving Eqs. (4) in the text. (It is a complex expression, but evaluates to a real number for parameter values used in this study).
(0.03 MB PDF)
