# Supplementary figures and images for: Model of Transcriptional Activation by MarA in Escherichia coli
Source: PLoS Comput Biol. 2009 Dec 18;5(12):e1000614. doi: 10.1371/journal.pcbi.1000614 (PMC2787020; doi:10.1371/journal.pcbi.1000614)

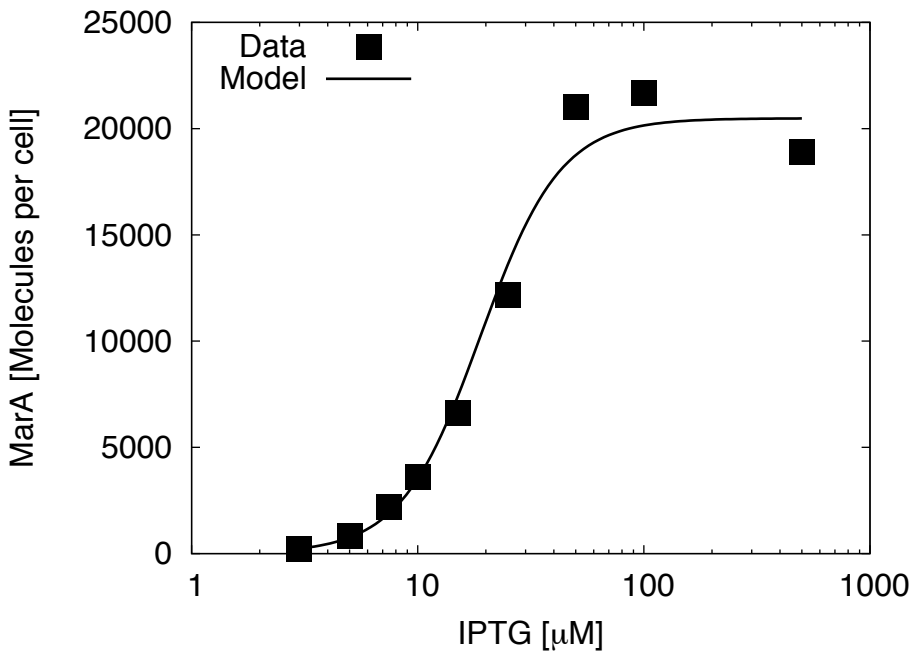

Supplement: Figure S1 — Calibration of IPTG levels against MarA levels. The data (boxes) are well-described by Eq. (6) (line). (0.02 MB PDF) [file pcbi.1000614.s001.pdf]

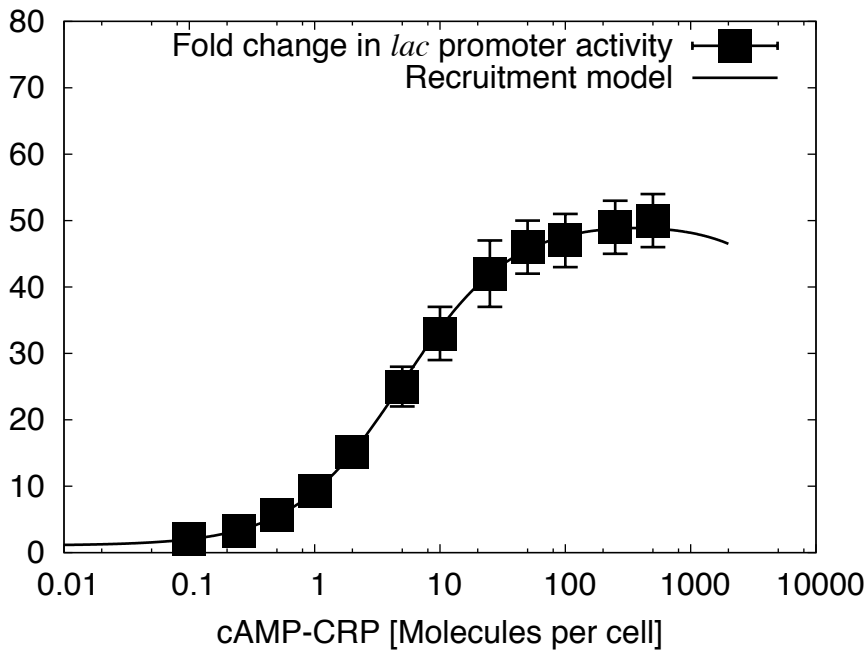

Supplement: Figure S2 — Fit of the recruitment model to CRP-dependent activity of the lac promoter. The data were generated using a Hill equation based on previously measured promoter activity data from Ref. [15], and the error bars were arbitrarily assigned for the fitting. (0.02 MB PDF) [file pcbi.1000614.s002.pdf]
