# Supplementary material for: Model of Transcriptional Activation by MarA in Escherichia coli
Source: PLoS Comput Biol. 2009 Dec 18;5(12):e1000614. doi: 10.1371/journal.pcbi.1000614 (PMC2787020; doi:10.1371/journal.pcbi.1000614)
Supplement: Table S1 — Promoter activity data. (0.07 MB PDF) [file pcbi.1000614.s003.pdf]

IPTG [μM]	*marRAB* Mean	*marRAB* Error[a]	*sodA* Mean	*sodA* Error[a]	*micF* Mean	*micF* Error[a]
0			1124	98		
0.1	1247	29	1012	45		
0.25	1298	28	1010	79	171	29
0.5	1409	57	1050	74	177	28
1	1663	40	1119	79	175	35
2	2238	109	1196	84	175	26
5	2352	72	2025	256	367	37
10	2466	125	2120	194	714	57
25	2565	98	2701	248	826	59
50	2601	122	2737	237	824	42
100	2590	112	2895	165	816	45
250	2524	110	2936	207	813	32
500	2397	135	2970	365	798	17

[a]Standard error of the mean $\sigma/\sqrt{N}$ calculated from N measurements.
