# Supplementary material for: Model of Transcriptional Activation by MarA in Escherichia coli
Source: PLoS Comput Biol. 2009 Dec 18;5(12):e1000614. doi: 10.1371/journal.pcbi.1000614 (PMC2787020; doi:10.1371/journal.pcbi.1000614)
Supplement: Text S1 — Equation for the free polymerase concentration obtained by solving Eqs. (4) in the text. (It is a complex expression, but evaluates to a real number for parameter values used in this study). (0.03 MB PDF) [file pcbi.1000614.s004.pdf]

$$
R = 1/3 (-AT - BT - KAR - KBR + RT) - ((1 + \mathbf{i}\, 3^{(1/2)}) (-(-AT - BT - KAR - KBR + RT)^2 - 3 (-BT\, KAR - AT\, KBR - KAR\, KBR + KAR\, RT + KBR\, RT)))/(3\, 2^{(2/3)} (2 AT^3 + 6 AT^2 BT + 6 AT\, BT^2 + 2 BT^3 + 6 AT^2 KAR + 3 AT\, BT\, KAR - 3 BT^2 KAR + 6 AT\, KAR^2 - 3 BT\, KAR^2 + 2 KAR^3 - 3 AT^2 KBR + 3 AT\, BT\, KBR + 6 BT^2 KBR - 6 AT\, KAR\, KBR - 6 BT\, KAR\, KBR - 3 KAR^2 KBR - 3 AT\, KBR^2 + 6 BT\, KBR^2 - 3 KAR\, KBR^2 + 2 KBR^3 - 6 AT^2 RT - 12 AT\, BT\, RT - 6 BT^2 RT - 3 AT\, KAR\, RT + 6 BT\, KAR\, RT + 3 KAR^2 RT + 6 AT\, KBR\, RT - 3 BT\, KBR\, RT - 12 KAR\, KBR\, RT + 3 KBR^2 RT + 6 AT\, RT^2 + 6 BT\, RT^2 - 3 KAR\, RT^2 - 3 KBR\, RT^2 - 2 RT^3 + \surd((2 AT^3 + 6 AT^2 BT + 6 AT\, BT^2 + 2 BT^3 + 6 AT^2 KAR + 3 AT\, BT\, KAR - 3 BT^2 KAR + 6 AT\, KAR^2 - 3 BT\, KAR^2 + 2 KAR^3 - 3 AT^2 KBR + 3 AT\, BT\, KBR + 6 BT^2 KBR - 6 AT\, KAR\, KBR - 6 BT\, KAR\, KBR - 3 KAR^2 KBR - 3 AT\, KBR^2 + 6 BT\, KBR^2 - 3 KAR\, KBR^2 + 2 KBR^3 - 6 AT^2 RT - 12 AT\, BT\, RT - 6 BT^2 RT - 3 AT\, KAR\, RT + 6 BT\, KAR\, RT + 3 KAR^2 RT + 6 AT\, KBR\, RT - 3 BT\, KBR\, RT - 12 KAR\, KBR\, RT + 3 KBR^2 RT + 6 AT\, RT^2 + 6 BT\, RT^2 - 3 KAR\, RT^2 - 3 KBR\, RT^2 - 2 RT^3)^2 + 4 (-(-AT - BT - KAR - KBR + RT)^2 - 3 (-BT\, KAR - AT\, KBR - KAR\, KBR + KAR\, RT + KBR\, RT))^3))^{(1/3)}) + (1/(6\, 2^{(1/3)}))(1 - \mathbf{i}\, 3^{(1/2)}) (2 AT^3 + 6 AT^2 BT + 6 AT\, BT^2 + 2 BT^3 + 6 AT^2 KAR + 3 AT\, BT\, KAR - 3 BT^2 KAR + 6 AT\, KAR^2 - 3 BT\, KAR^2 + 2 KAR^3 - 3 AT^2 KBR + 3 AT\, BT\, KBR + 6 BT^2 KBR - 6 AT\, KAR\, KBR - 6 BT\, KAR\, KBR - 3 KAR^2 KBR - 3 AT\, KBR^2 + 6 BT\, KBR^2 - 3 KAR\, KBR^2 + 2 KBR^3 - 6 AT^2 RT - 12 AT\, BT\, RT - 6 BT^2 RT - 3 AT\, KAR\, RT + 6 BT\, KAR\, RT + 3 KAR^2 RT + 6 AT\, KBR\, RT - 3 BT\, KBR\, RT - 12 KAR\, KBR\, RT + 3 KBR^2 RT + 6 AT\, RT^2 + 6 BT\, RT^2 - 3 KAR\, RT^2 - 3 KBR\, RT^2 - 2 RT^3 + \surd((2 AT^3 + 6 AT^2 BT + 6 AT\, BT^2 + 2 BT^3 + 6 AT^2 KAR + 3 AT\, BT\, KAR - 3 BT^2 KAR + 6 AT\, KAR^2 - 3 BT\, KAR^2 + 2 KAR^3 - 3 AT^2 KBR + 3 AT\, BT\, KBR + 6 BT^2 KBR - 6 AT\, KAR\, KBR - 6 BT\, KAR\, KBR - 3 KAR^2 KBR - 3 AT\, KBR^2 + 6 BT\, KBR^2 - 3 KAR\, KBR^2 + 2 KBR^3 - 6 AT^2 RT - 12 AT\, BT\, RT - 6 BT^2 RT - 3 AT\, KAR\, RT + 6 BT\, KAR\, RT + 3 KAR^2 RT + 6 AT\, KBR\, RT - 3 BT\, KBR\, RT - 12 KAR\, KBR\, RT + 3 KBR^2 RT + 6 AT\, RT^2 + 6 BT\, RT^2 - 3 KAR\, RT^2 - 3 KBR\, RT^2 - 2 RT^3)^2 + 4 (-(-AT - BT - KAR - KBR + RT)^2 - 3 (-BT\, KAR - AT\, KBR - KAR\, KBR + KAR\, RT + KBR\, RT))^3))^{(1/3)} \tag{S1}
$$
